# A Theoretical Study on the Electronic Excitation of the Pyridine Molecule by Electron Impact

**DOI:** 10.1021/acsphyschemau.5c00094

**Published:** 2025-11-18

**Authors:** Murilo O. Silva, Márcio H. F. Bettega, Romarly F. da Costa

**Affiliations:** † Instituto Federal do Paraná, Campus Avançado Goioerê, Rodovia Luiz Dechiche, s/no, 87360-000 Goioerê, Curitiba, Paraná, Brazil; ‡ Departamento de Física, 28122 Universidade Federal do Paraná, Caixa Postal 19044, 81531-980 Curitiba, Paraná, Brazil; § Centro de Ciências Naturais e Humanas, 74362 Universidade Federal do ABC, 09210-580 Santo André, São Paulo, Brazil

**Keywords:** electron scattering, electronic excitation, Schwinger multichannel method, multichannel coupling, pyridine.

## Abstract

In this work, we present a theoretical investigation of electron collisions by the pyridine molecule. Elastic cross sections and electronic inelastic cross sections involving the transitions from the ground state to the 1^3^ *A*_1_, 1^3^ *B*_2_, 2^3^ *A*_1_, 1^3^ *B*_1_, 1^3^ *A*_2_, 1^1^ *B*_2_, 1^1^ *B*_1_, and 1^1^ *A*_2_ excited states of pyridine are reported in the energy range from 0 to 50 eV. The scattering amplitudes were obtained using the Schwinger multichannel method, and the effects of multichannel coupling were accounted for by means of the minimal orbital basis for single-configuration interactions strategy. This strategy gives rise to an up to 301-states level of channel coupling calculation and enables us to evaluate the influence of flux stealing due to competition of energetically accessible channels upon the magnitude of the cross sections. Our computed elastic cross sections are in very good agreement with existing experimental data and provide positions for the three π* resonances, which are consistent with previous assignments. The present results involving the transitions from the ground state to the lowest-lying excited states of the pyridine are shown to be very sensitive to the influence of opening thresholds. Compared with the only theoretical result reported in the literature so far, our excitation cross sections present a higher magnitude. Despite this fact, for almost all transitions considered, the agreement in terms of general trends is reasonable and quite encouraging.

## Graphical abstract

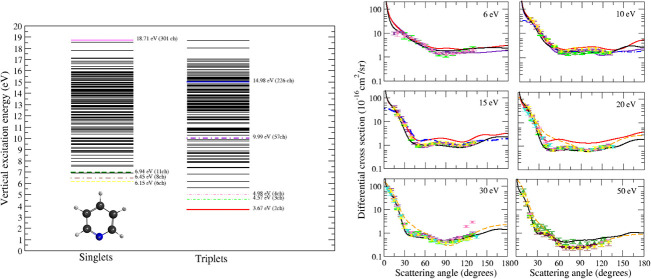

## 1 Introduction

The study of low-energy electron scattering by biologically relevant molecules has received significant attention since the pioneering work of Boudaïffa et al.,^1^ who experimentally demonstrated that low-energy electrons produced by ionizing radiation can induce lethal damage to DNA in the form of single- and double-strand breaks. In particular, the findings obtained in this work brought to light the fact that these types of damage eventually occur when electrons are temporarily trapped in unoccupied molecular orbitals, forming resonant states that can lead to bond cleavage. In this context, both theoretical and experimental efforts have focused on understanding the mechanisms by which low-energy electrons interact with the molecular components of the biological environment. Such studies encompass investigations involving nucleobases,^2–4^ sugar structures,^5^ as well as simple molecules that serve as prototypes^6^ for the subsequent study of more complex systems. In line with this approach, we present a study on electron scattering by pyridine, whose molecular structure can be seen in Figure 1, a molecule of particular interest due to its structural similarity to the nitrogenous bases of DNA. The electron scattering data obtained for this system are especially relevant for modeling electron-induced damage in biomolecular environments.^7^ In fact, accurate electron scattering cross sections play a fundamental role in modeling radiation-induced damage in biological systems, as they serve as key inputs for Monte Carlo simulations that trace electron paths and estimate how radiation interacts with matter.^8,9^ These simulations are widely employed in radiobiology, medical physics, and radiation therapy planning, where understanding the spatial and energy distribution of secondary electrons is crucial for dose assessment at the molecular level.^10,11^ Consequently, a comprehensive understanding of the behavior of low-energy electrons, typically below 20 eV, and their chemical interactions within living cells is vital for advancing research areas involving radiation, such as cancer radiotherapy, radiation protection, and space biology.

**Figure 1:**
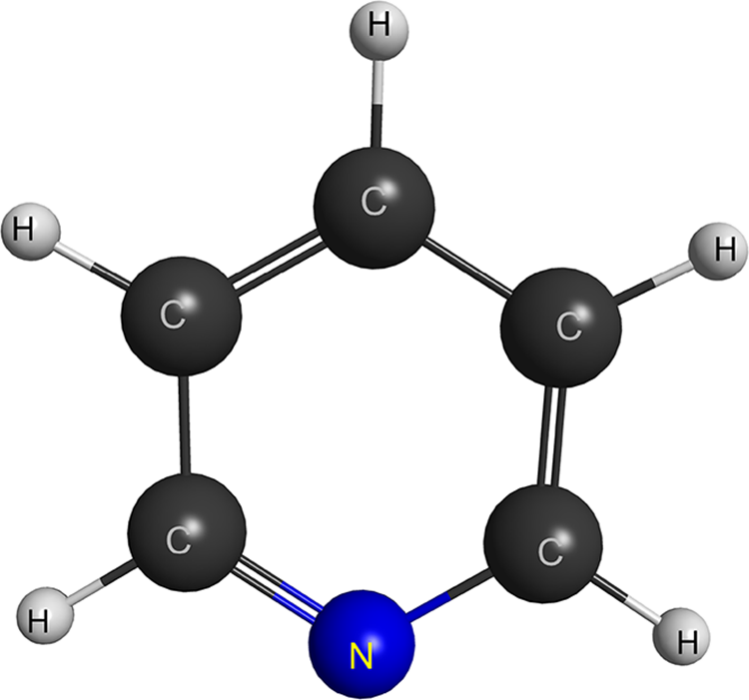
Ball and stick model of the pyridine molecule generated with MacMolPlt.^57^

Owing to its similarity with DNA basic components, in particular the pyrimidinic systems, pyridine has been the subject of extensive investigation by theoretical and experimental groups. Nenner and Schulz^12^ were pioneers in the study of electron-pyridine interactions, using the electron transmission spectroscopy (ETS) technique to investigate the formation of temporary negative ions. In their work, the authors identified three resonances centered at 0.62, 1.20, and 4.58 eV. The first two resonances were characterized as being of π* type, and Nenner and Schulz^12^ suggested that the third one exhibited a mixed nature, combining features of a shape resonance with those of a core-excited resonance. Later, Mathur and Hasted^13^ also employed ETS and observed these same resonances, although at the slightly different values of 0.79, 1.15, and 4.71 eV. Similarly, Modelli and Burrow^14^ applied the ETS technique and detected resonances at 0.72, 1.18, and 4.48 eV. Using the Schwinger multichannel (SMC) method, Barbosa et al.^15^ reported theoretical results for electron scattering by the pyridine molecule at energies up to 12 eV. Their calculations were performed at two levels of approximation—static exchange (SE) and static exchange plus polarization (SEP)—considering only the elastic channel as open. In this work, the authors identified the same three resonances previously reported in the literature and located at 0.90, 1.33, and 5.80 eV in the calculations performed at the SEP level of approximation. Complementing these studies, Costa et al.^16^ investigated the electron scattering by pyridine in the 0 to 100 eV energy range, providing a comprehensive set of differential and integral cross sections for both elastic and inelastic processes, including rotational, vibrational, and electronic excitations, as well as ionization. The data were obtained through a combined approach, involving theoretical calculations and experimental measurements. Experimental cross sections were determined using the electron transmission technique and the reaction microscope coincidence analysis.^16^ To meet the requirements of transport models, theoretical cross sections were computed using the independent atom model with screening corrected additivity rule and interference effects (IAM-SCAR) method for energies above 10 eV, while R-matrix and SMC with pseudopotential methods were applied below 15 and 20 eV, respectively. The resonance positions observed in this work were 0.77, 1.11, and 5.51 eV. More recently, Su et al.^17^ calculated total cross sections (TCS) for the electron scattering by pyridine, encompassing both elastic and inelastic processes. The authors employed two distinct approximations: static-exchange plus polarization (SEP) and close-coupling (CC). The calculated resonance positions were 0.61, 1.10, and 5.35 eV for the SEP approximation and 0.82, 1.07, and 5.66 eV for the CC approximation. Moreover, the authors employed a time-delay analysis to characterize the core-excited resonances. Dubuis et al.^18^ reported experimental measurements of TCS for electron scattering by the pyridine molecule in the energy range of 10 to 1000 eV, with uncertainties between 5% and 10%, using an apparatus with double-electrostatic analyzers. Complementarily, Lozano et al.^19^ investigated the TCS of pyridine for impact energies between 1 and 200 eV, employing a magnetically confined electron-beam system, with particular attention to energies below 10 eV. They evaluated systematic errors associated with the detector acceptance angle for elastically and rotationally inelastically scattered electrons, and their data showed good agreement with previous measurements above 10 eV. Finally, Szmytkowski et al.^20^ measured the absolute total cross section for energies from 0.6 to 300 eV in a linear electron transmission experiment, observing an energy dependence typical of targets with a high electric dipole moment. They identified a broad enhancement centered around 8.5 eV in the 3 to 20 eV range as well as weak structures below 10 eV attributed to resonant scattering processes.

In the present study, we report on integral, differential, and total cross sections for the elastic and electronically inelastic scattering of electrons by the pyridine molecule in the energy range from 0 to 50 eV. The calculations were performed using the SMC method implemented with pseudopotentials.^21,22^ To account for multichannel coupling effects, we applied the minimal orbital basis for single-configuration interactions (MOB-SCI) strategy,^23^ considering between 1 and 301 open channels in the scattering calculations. Cross sections were obtained for excitations from the ground state to the triplet 1^3^ *A*_1_, 1^3^ *B*_2_, 2^3^ *A*_1_, 1^3^ *B*_1_, and 1^3^ *A*_2_ states, as well as to the singlet 1^1^ *B*_2_, 1^1^ *B*_1_, and 1^1^ *A*_2_ states, and the results are compared with available literature data. To estimate the TCS, we combined the elastic and electronically inelastic contributions obtained with the SMC method with those from the binary-encounter-Bethe (BEB) model.^24^ This hybrid approach has already been successfully employed in previous studies conducted by our research group.^6,25,26^

The structure of this article is as follows: Section 2 outlines the theoretical framework employed in the present calculations, while Section 3 describes the computational details of the bound states and scattering calculations. The results and discussions are presented in Section 4. Finally, the main conclusions are summarized in Section 5.

## 2 Methods

The elastic and electronically inelastic cross sections presented in this work were obtained using the SMC method,^21,22^ implemented with the norm-conserving pseudopotentials of Bachelet, Hamann, and Schlüter (BHS).^27^ These pseudopotentials represent the nuclei and core electrons of C and N through a smooth potential that correctly reproduces the valence states. The SMC method is an extension of Schwinger’s variational principle, developed to obtain an expression for the scattering amplitude. It incorporates effects that are essential in the description of electron-molecule interactions, such as exchange interaction, polarization effects, and the competition between elastic and electronically inelastic channels through the inclusion of multichannel coupling effects, in an *ab initio* fashion. Below, we highlight key aspects of the method. For a more detailed description, refer to ref 23, where the method is reviewed.

In the SMC method, the working expression obtained for the scattering amplitude is given by

1 $$f\left({\mathrm{k⃗}}_{f},{\mathrm{k⃗}}_{i}\right)=-\frac{1}{2\pi }\sum_{m,n}\left\langle S_{{\mathrm{k⃗}}_{f}}\right|V\left|\chi_{m}\right\rangle {\left(d^{-1}\right)}_{\mathrm{mn}}\left\langle \chi_{n}\right|V\left|S_{{\mathrm{k⃗}}_{i}}\right\rangle$$

where

2 $$d_{\mathrm{mn}}=\left\langle \chi_{m}\right|A^{\left(+\right)}\left|\chi_{n}\right\rangle$$

and the operator *A* ^(+)^ is given by

3 $$A^{\left(+\right)}=\frac{Ĥ}{N+1}-\frac{\left(ĤP+PĤ\right)}{2}+\frac{\left(\mathrm{PV}+\mathrm{VP}\right)}{2}-VG_{P}^{\left(+\right)}V$$

In the equations above, |*S*_*k⃗*_*i*(*f*)__⟩ is an eigenstate of the unperturbed Hamiltonian *H*_0_ = *H*_*N*_ + *T*_*N*+1_, expressed as the product of a target state and a plane wave, where *k⃗*_*i*(*f*)_ represents the momentum of the free incident (scattered) electron. In the definition of *H*_0_, *H*_*N*_ denotes the target Hamiltonian, while *T*_*N*+1_ corresponds to the kinetic energy operator of the incident electron. The term *V* represents the interaction potential between the incident electron and the target’s electrons and nuclei. The operator *Ĥ* = *E* – *H* is defined in terms of *E*, the total collision energy, and *H*, the (*N*+1)-electron Hamiltonian under the fixed-nuclei approximation. Additionally, *G*_*P*_^(+)^ = *PG*_0_^(+)^ is the free-particle Green’s function projected onto the *P*-space, where *P* is a projection operator onto the open-channel space of the target, given by the following expression

4 $$P=\sum_{l=1}^{N_{\mathrm{open}}}\left|\Phi_{l}\right\rangle \left\langle \Phi_{l}\right|$$

where |Φ_*l*_⟩ represents the target states, which may correspond to the ground state (*l* = 1) or any electronically excited state (*l* ≥ 2) of the target molecule. As the incident electron energy increases, a great number of channels become energetically accessible. Thus, *N*_open_ represents the number of accessible channels treated as being open in the calculations.

The state |χ_*m*_⟩ represents a basis set of (*N* + 1)-electron Slater determinants, also known as configuration state functions (CSFs). These CSFs are constructed as spin-adapted products of target states and single-particle scattering orbitals

5 $$\left|\chi_{\mathrm{mn}}\right\rangle =A\left[\left|\Phi_{m}^{s}\right\rangle ⊗\left|\phi_{n}\right\rangle \right]$$

Here, A is the antisymmetrization operator and |Φ_*m*_^*s*^⟩ represents the molecular target state. The ground state, obtained at the Hartree–Fock level, is denoted by |Φ_1_^0^⟩, while |Φ_*m*_^*s*^ ⟩ (*m* ≥ 2) corresponds to an *N*-electron Slater determinant. This determinant is constructed by performing single excitations from the occupied valence (hole) orbitals of the ground (reference) state to a set of unoccupied (particle) orbitals with spin *s* (*s* = 0 for singlet states and *s* = 1 for triplet states). |ϕ_*n*_⟩ represents a scattering orbital.

To obtain the TCS, we combined the contributions from elastic and electronically inelastic cross sections, calculated using the SMC method, with the ionization cross section. For the ionization component, we employed the BEB model,^24^ which is widely used due to its simple analytical formulation for estimating total ionization cross section (TICS) resulting from electron collisions with atoms and molecules. In this approach, the ionization cross section for a given molecular orbital *i*-th is defined as

6 $$\sigma_{i}\left(t_{i}\right)=\frac{4\pi a_{0}^{2}N_{i}{\left(R/B_{i}\right)}^{2}}{t_{i}+u_{i}+1}\times \left[\frac{\ln\left(t_{i}\right)}{2}\left(1-\frac{1}{t_{i}^{2}}\right)+1-\frac{1}{t_{i}}-\left(\frac{\ln\left(t_{i}\right)}{t_{i}+1}\right)\right]$$

where *B*_*i*_ is the binding energy of the electron at the *i*-th molecular orbital, *t*_*i*_ = *E*/*B*_*i*_, *u*_*i*_ = *U*_*i*_/*B*_*i*_, where *E* is the kinetic energy of the incident electron, *U*_*i*_ is the average kinetic energy of the *i*-th molecular orbital, *a*_0_ is the Bohr radius, *R* is the Rydberg energy, and *N*_*i*_ is the occupation number of the *i*-th molecular orbital. The TICS is determined by adding up the ionization cross sections of all orbitals participating in the process, namely

7 $$\sigma_{\mathrm{BEB}}=\sum_{i=1}^{N_{\mathrm{occ}}}\sigma_{i}\left(t_{i}\right)$$

Here, *N*_occ_ represents the number of occupied molecular orbitals in the target molecule. All parameters appearing in the above definitions for the ionization cross sections were derived from a Hartree–Fock calculation performed at the equilibrium geometry in the ground state, using the aug-cc-pVDZ basis set within the GAMESS^28^ computational package. The calculated ionization threshold was 9.42 eV, showing good agreement with the experimental value of 9.26 eV.^29^

## 3 Computational Details

The geometry of the ground state of pyridine was optimized in the C_2*v*_ point group at the second-order Møller–Plesset (MP2) theory, employing the aug-cc-pVDZ basis set, through the GAMESS^28^ computational package. The nuclei and core electrons of carbon and nitrogen atoms are represented by the BHS pseudopotentials, while the valence electrons are described using a set of 5s5p2d uncontracted Cartesian Gaussian (CG) functions, generated following the procedure outlined in ref. 30. Table 1 lists the exponents of the CG functions used in this work. For the hydrogen atoms, we employed the Dunning^31^ 4s/3s basis set, supplemented by an additional *p*-type function with an exponent of 0.75. The ground state of the target molecule is determined at the Hartree–Fock level, while the excited states are described according to the MOB-SCI^32^ strategy. More precisely, as detailed below, all electronically excited states are initially obtained through a full single-configuration interaction (FSCI) calculation. Then, for the excited states under study, among all hole-particle pairs, only those with the most significant contributions (greater statistical weight in the composition of a given excited state) are selected. This procedure gives rise to a second spectrum, which, in turn, underpins the construction of the MOB-SCI scheme.

**Table 1:** Exponents of the Uncontracted Cartesian Gaussian Functions Used for Carbon (C) and Nitrogen (N) Atoms in the Present Calculations Performed with the SMC Method

type	C	N
s	12.49628	17.56734
s	2.470286	3.423615
s	0.614028	0.884301
s	0.184028	0.259045
s	0.039982	0.055708
p	5.228869	7.050692
p	1.592058	1.910543
p	0.568612	0.579261
p	0.210326	0.165395
p	0.072250	0.037192
d	0.603592	0.403039
d	0.156753	0.091192

The procedure for the scattering calculations followed these steps: (i) first, we used improved virtual orbitals (IVOs)^33^ to represent the particle and scattering orbitals. With these orbitals in hand, we performed a FSCI calculation in which, for the pyridine molecule, 3105 singly excited Slater determinants (or 3105 hole-particle pairs) were obtained. This process resulted in a total of 6210 electronically excited states, comprising 3105 singlet and 3105 triplet states; (ii) From these 6210 states, we selected the 200 lowest-energy electronically excited states, which will be used as our reference states. To describe these 200 states, we employed 150 hole-particle pairs (more specifically, as mentioned before, the pairs with the highest contributions for the selected states), resulting in 300 electronically excited states, of which 150 are singlets and 150 are triplets. When choosing the hole-particle pairs for the MOB-SCI scheme, we ensured that the energy values obtained by using this approach maintained at least a 90% level of agreement with those derived from the FSCI calculation.

In Table 2, we present the spectrum of the first 24 electronically excited states obtained from the FSCI calculation along with the results from the MOB-SCI strategy. An excellent agreement is observed between these two spectra, with the smallest difference being 0.02 eV and the largest difference being 0.74 eV. We also compared our results with the experimental^34–36^ and theoretical^17,34,37,38^ data available in the literature. We observed that, in some cases, there is an inversion of states, such as, for example, of our second triplet state 1^3^ *B*_2_, which corresponds to the fourth state in ref 17. Despite these inversions, our results show a good level of agreement with the data in the literature. The same hole-particle pairs used to define the active space in the MOB-SCI strategy were also employed to construct the CSF space. As a result, we obtained 14499 CSFs for symmetry *A*_1_, 14420 for symmetry *B*_1_, 15019 for symmetry *B*_2_, and 14875 for symmetry *A*_2_. As the incident electron energy increases, more channels become energetically accessible, thereby leading to a larger number of states being considered in the projection operator *P*. Consequently, different coupling levels are considered in our calculations as the energy increases. However, in this work, we present only the best coupling level for each specific energy. To distinguish between different coupling levels, we adopt the nomenclature used in ref 39, denoted as *N*_open_ch, where *N*_open_ represents the number of open channels considered in the *P* operator. In Figure 2, we present the spectrum of electronically excited states obtained using the MOB-SCI strategy, along with the different multichannel coupling levels considered in the scattering calculations, where the colored lines represent the various multichannel coupling levels employed in the scattering calculations. The coupling levels included are 2ch (3.67 eV), 3ch (4.57 eV), 4ch (4.98 eV), 6ch (6.15 eV), 8ch (6.45 eV), 11ch (6.94 eV), 57ch (9.99 eV), 226ch (14.98 eV), and 301ch (18.71 eV).

**Figure 2:**
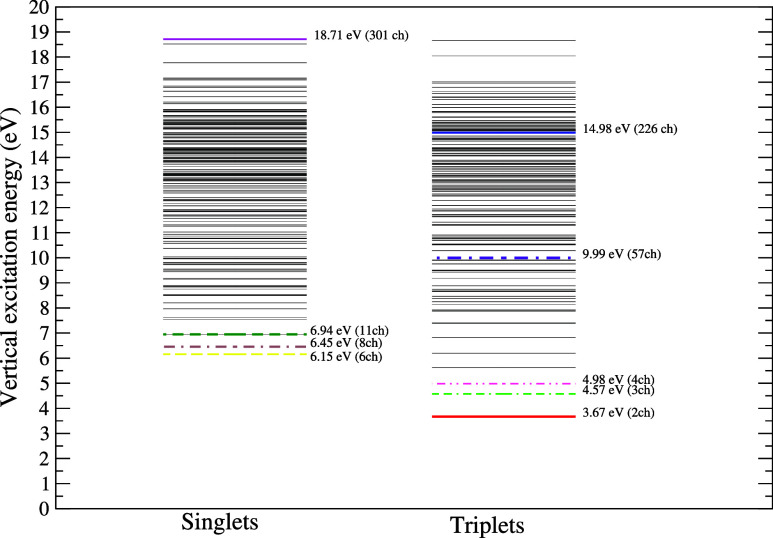
Schematic representation of the vertical excitation energies (in eV) of the 300 electronically excited states of pyridine obtained with the MOB-SCI calculation and different levels of channel coupling employed in the present scattering calculations performed by means of the SMC method. Solid red line, 2ch; double-dashed-dotted green line, 3ch; double-dotted-dashed pink line, 4ch; dashed yellow line, 6ch; dashed-dotted brown line, 8ch; dashed dark green line, 11ch; dotted-dashed purple line, 57ch; solid blue line, 226ch; solid magenta line, 301ch.

**Table 2:** Vertical Excitation Energies (in eV) for the First 24 Excited Electronic Singlets and Triplets States Obtained from FSCI and MOB-SCI Calculations.^a^

state	FSCI	MOB-SCI	ref 17	ref 37	ref 38	ref 34	ref 35	ref 36
1^3^ *A*_1_	3.27	3.67	4.64	4.05	4.06	3.86		4.10
1^3^ *B*_2_	4.48	4.57	5.55	4.56	4.64	4.47		4.84
2^3^ *A*_1_	4.83	4.98	5.62	4.73	4.91			4.84
1^3^ *B*_1_	4.96	5.61	5.19	4.41	4.25	4.12		4.10
1^1^ *B*_2_	5.96	6.15	5.96	4.84	4.85	4.99	4.99	
2^3^ *B*_2_	6.00	6.19	6.85	6.02	6.08	6.09		
1^1^ *B*_1_	6.01	6.45	5.83	4.91	4.59	4.78	4.74	
2^1^ *A*_1_	6.30	6.93	7.65	6.70	6.17	6.30	6.28	6.30
1^3^ *A*_2_	6.76	6.82	6.29	5.10	5.28	5.40		5.40
1^1^ *A*_2_	6.91	6.94	6.38	5.17	5.11	5.74		5.43
2^3^ *A*_2_	7.05	7.43						
2^1^ *A*_2_	7.31	7.61						
2^3^ *B*_1_	7.31	7.37						
3^3^ *A*_1_	7.45	7.89		7.34				
2^1^ *B*_1_	7.50	7.53						
3^3^ *B*_1_	7.80	7.87						
3^3^ *A*_2_	7.90	7.92						
3^1^ *B*_1_	7.92	7.96						
3^1^ *A*_2_	7.94	7.96						
3^3^ *B*_2_	7.96	8.14		7.28				
2^1^ *B*_2_	8.07	8.2		7.48	7.27	8.30	7.22	7.20
4^3^ *B*_2_	8.12	8.24						
3^1^ *A*_1_	8.13	8.87		6.42	6.26	6.39	6.38	

a We compared our results with theoretical data reported by Su et al.,^17^ who employed the state-averaged complete active space self-consistent field (SA-CASSCF) method; by Lorentzon et al.,^37^ who used the complete active space self-consistent field (CASSCF) method; and by Wan et al.,^38^ who applied the symmetry-adapted cluster configuration interaction (SAC–CI) approach. Additionally, we compared our findings with experimental data obtained by Linert and Zubek,^34^ using electron energy loss spectroscopy (EELS), as well as with the measurements reported by Bolovinos et al.^35^ and Walker et al.,^36^ who employed both vacuum ultraviolet (VUV) absorption spectroscopy and EELS techniques.

The pyridine molecule is polar, with a calculated permanent dipole moment of 2.41 D, in good agreement with the experimental^29^ value of 2.19 D. This dipole moment generates a long-range interaction potential, significantly influencing the scattering process, particularly at low energies and small angles. In calculations carried out with the SMC method, the long-range effects of the potentials are truncated due to the use of square-integrable (*L* ^2^) functions in the description of the scattering states. To mitigate this limitation, we applied the Born-closure procedure, which combines the scattering amplitude obtained from the SMC method with the scattering amplitude due to the dipole potential calculated by using the First Born Approximation (FBA). In summary, the scattering amplitude derived from the SMC method is expanded in partial waves up to a specific value, *l*_SMC_, while the dipole potential scattering amplitude is computed using the FBA and similarly expanded in partial waves. These two amplitudes are then merged: the SMC amplitude governs partial waves up to *l*_SMC_, whereas the dipole amplitude accounts for contributions beyond *l*_SMC+1_ and extends to ∞. The appropriate *l*_SMC_ value is determined by comparing the DCSs obtained with and without the Born-closure correction, which align with each other for scattering angles greater than approximately 20°. Additional methodological details can be found in ref 23. The selection of *l*_SMC_ values depends on the incident electron energy. The chosen values were: *l* = 1 for the interval from 0.1 to 1.4 eV, *l* = 2 from 1.0 to 1.5 eV, *l* = 3 from 1.5 to 2.0 eV, *l* = 4 from 2.5 to 3.0 eV, *l* = 5 from 3.5 to 3.9 eV, *l* = 6 from 4.0 to 4.9 eV, *l* = 7 from 5.0 to 7.9 eV, *l* = 8 from 8.0 to 8.5 eV, *l* = 8.6 from 9.5 to 9.9 eV, and *l* = 10 from 10.0 to 50.0 eV.

## 4 Results and Discussion

### 4.1 Elastic Cross Section

In Figure 3, we present the elastic integral (ICS) and total cross sections for electron scattering by the pyridine molecule for impact energies of up to 50 eV, as obtained in the present work. Our elastic ICS was calculated considering 1 to 301 open channels, applying the Born-closure procedure to correct for the long-range potential due to the molecular dipole moment. Present results reveal the presence of three structures located at 0.70, 1.60, and 5.00 eV. The first two have been reported in the literature^15^ as π*shape resonances, while the third exhibits a mixed character of shape and core-excited resonance. Due to the presence of other structures in this energy region, we performed a diagonalization of the scattering Hamiltonian to confirm the position of the third resonance. We obtained an eigenvalue of 5.09 eV, which supports the presence of the structure observed in our scattering calculations at 5.00 eV. With regard to the other peaks that appear in this energy region, it is worth noting that structures of this kind can be interpreted as manifestations of threshold effects, in which the opening of a new inelastic channel leads to a sudden change in the cross section. Such behavior has been reported in previous studies involving collisions not only with electrons,^40,41^ but also with other particles.^42–45^ This phenomenon, known in the literature as the Wigner-cusp effect,^46^ is related to the unitarity and analyticity of the scattering matrix near the threshold for the opening of a new channel. As a consequence, the cross section may exhibit a discontinuity or a cusp-like anomaly at that point.

**Figure 3:**
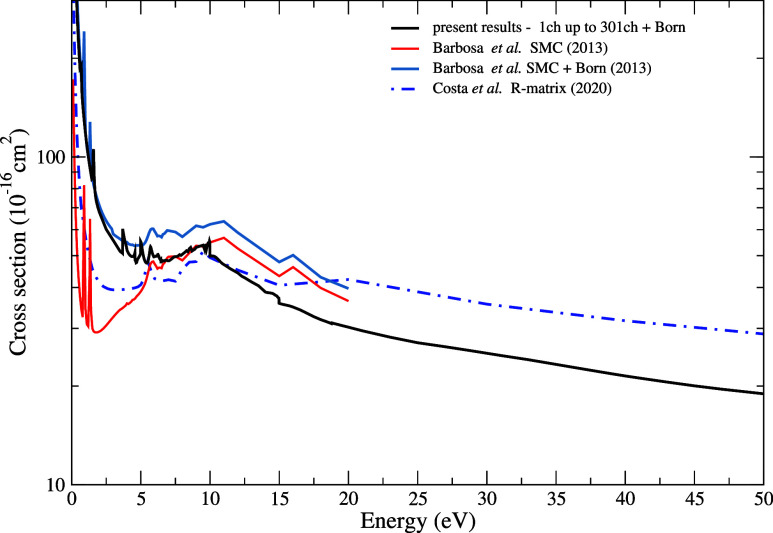
Elastic cross section for electron scattering by the pyridine molecule for impact energies up to 50 eV. Black solid line, present elastic ICS considering 1 to 301 open channels with the Born-closure procedure; red and steel blue solid lines, elastic ICS obtained by Barbosa et al.^15^ using the SMC method, without and with the Born-closure correction, respectively; blue dashed-dotted line, elastic ICS obtained by Costa et al.^16^ using the R-matrix method.

For the first resonance, our results are in agreement with the positions measured by Nenner and Schulz,^12^ Modelli and Burrow,^14^ and Mathur and Hasted,^13^ who observed the resonance formation at 0.62, 0.72, and 0.79 eV, respectively. The second resonance is located at a slightly higher energy than the experimental values (1.20 eV for Nenner and Schulz,^12^ 1.18 eV for Modelli and Burrow,^14^ and 1.15 eV for Mathur and Hasted^13^), but still within a reasonable margin of agreement, with a difference of around 0.40 eV. Similarly, the third resonance also shows a reasonable agreement with the experimental data (4.58 eV for Nenner and Schulz,^12^ 4.48 eV for Modelli and Burrow,^14^ and 4.71 eV for Mathur and Hasted^13^). Barbosa et al.^15^ reported theoretical results obtained using the SMC method, with and without the dipole moment correction, considering only the open elastic channel. Our results show good agreement with the results of Barbosa et al.^15^ up to approximately 6 eV. Above this energy range, our calculations included more open channels, leading to a reduction in the magnitude of the cross section due to the competition between elastic and electronically inelastic channels for the flux that defines the cross section. Regarding the position of the resonant structures, the first resonance in our study is 0.20 eV below the one reported by Barbosa et al.,^15^ while the second is 0.27 eV above. The third, however, is 0.80 eV below the value obtained by Barbosa et al.,^15^ indicating that the first and second resonances are in good agreement with their findings. For the results obtained using the R-matrix method by Costa et al.,^16^ our cross section values are higher than those reported by these authors for energies below 10 eV. For energies above 10 eV, a good agreement between the results is observed. Su et al.^17^ discussed the discrepancies between the elastic scattering calculations performed by Barbosa et al.^15^ and Costa et al.,^16^ who used the SMC and R-matrix methods, respectively. According to Su et al.,^17^ the main differences in the magnitudes of the cross sections can be attributed to the distinct electronic distribution in the *L* ^2^ configurations and the different number of partial waves considered. In the R-matrix method, the value of *l*_max_ = 4 is adopted throughout the entire energy range, whereas in the SMC method, this limit is higher. Furthermore, the angular integration range may influence the observed discrepancies, especially in the results that include Born corrections, being one of the possible explanations for the difference between our results and the R-matrix results obtained by Costa et al.^16^

Su et al.^17^ presented theoretical results at two levels of calculation, SEP and CC, providing the TCS. At this point, we will discuss only the positions of the resonances; the TCS obtained by these authors will be analyzed later in comparison with our results. The authors highlight that shape resonances are better described in the SEP calculation, while those with mixed or core-excited character are better represented by the CC calculation. In their SEP calculation, the first and second resonances appear, respectively, 0.07 and 0.49 eV below the positions identified in our work, while the third resonance occurs 0.35 eV above our result. In the CC calculation, the first resonance reported by these authors is 0.12 eV above the value identified in our study, the second is 0.52 eV below, and the third is 0.66 eV above our results. Overall, the resonance positions obtained in our work are in reasonable agreement with the results presented by Su et al.^17^ The positions of the resonances are presented in Table 3.

**Table 3:** Comparison between the Positions of the Resonances (in eV) Observed in the Elastic Scattering of Electrons by the Pyridine Molecule

	π_1_^*^	π_2_^*^	π_3_^*^
theoretical results			
present results	0.70	1.60	5.00
Barbosa et al.^15^	0.90	1.33	5.80
Costa et al.^16^	0.77	1.11	5.51
Su et al.^17^ (SEP)	0.61	1.10	5.35
Su et al.^17^ (CC)	0.82	1.07	5.66
experimental results			
Nenner and Schulz^12^	0.62	1.20	4.58
Modelli and Burrow^14^	0.72	1.18	4.48
Mathur and Hasted^13^	0.79	1.15	4.71

In Figure 4, we present the DCS for electron scattering by the pyridine molecule at impact energies of 6, 10, 15, 20, 30, and 50 eV. We compare our results with the theoretical data of Barbosa et al.^15^ (SMC method), Costa et al.^16^ (IAM+SCAR and R-matrix methods), and Su et al.^17^ (R-matrix method). Since no experimental results are available in the literature for electron scattering by the pyridine molecule, we chose to compare our results with those for structurally similar molecules, such as benzene,^47–50^ pyrimidine,^51–53^ and pyrazine.^54^ The results obtained by Barbosa et al.^15^ using the SMC method, as mentioned before, considered only the elastic channel as open. At 6 eV, there is a difference in both the magnitude and oscillatory pattern of the cross section compared with our results. This occurs because, in this energy range, our calculations include four open channels in addition to the presence of some resonant structures. For impact energies of 10, 15, and 20 eV, although the overall behavior is similar, there is a significant difference in terms of magnitude. For these energies, we considered 57, 226, and 301 open channels, respectively, which drastically reduce the magnitude of the cross section. Costa et al.^16^ highlighted in their work that the DCSs for energies below 15 eV are obtained using the R-matrix method, while for higher energies, they used the IAM+SCAR method, as each approach provides a better description for the effects of the electron-pyridine interaction at the low and high energy range, respectively. In general, at 10 eV, the R-matrix results presented by the authors show reasonable agreement with ours, although differences in the oscillatory pattern are observed. This may be explained by the presence of structures in our calculations in this energy range. At 15 eV, we observed a similar behavior but with a difference in magnitude, attributed to the higher number of open channels included in our calculations. For the results of Costa et al.^16^ obtained with the IAM+SCAR method at 20, 30, and 50 eV, despite the similar magnitudes, we identified a difference in the oscillatory pattern, possibly due to other excitations considered by the authors in their calculations. The results obtained by Su et al.^17^ using the R-matrix method at 6 and 10 eV show a similar magnitude compared to ours but differ in the oscillatory behavior. Once again, this difference can be attributed to the presence of resonant structures in our calculations in these energy ranges. Our results show good agreement with the experimental data for the benzene molecule. At 6 eV, our results exhibit a similar behavior compared to the values obtained by Cho et al.^48^ for angles between 30° and 105°. At 10 eV, we observed good agreement for angles below 75° and above 90°, while in the intermediate range, there is a slight increase in the benzene cross section, though the oscillatory pattern remains the same. For 15, 20, and 30 eV, the results of Cho et al.^48^ show excellent agreement with ours. Compared to the data from Kato et al.^50^ and Sanches et al.^49^ at 50 eV, we observed a similarity in the oscillatory pattern of the cross sections but a difference in magnitude, with our results being higher than the experimental values. This discrepancy can be attributed to the fact that in this energy range more channels become accessible and are not considered in our calculations. Additionally, we emphasize that from this energy onward inclusion of the ionization channel becomes relevant for describing the electron scattering dynamics, making it a crucial factor for a more accurate comparison with the experimental data. The data obtained by Cadena et al.^47^ for energies between 10 and 50 eV show good agreement with our results, following the same pattern observed in other experimental data for the benzene molecule. Regarding the experimental electron scattering data for pyrimidine molecules obtained by Palihawadana et al.,^53^ Baek et al.^51^ and Maljković et al.,^52^ and pyrazine, obtained by Palihawadana et al.,^54^ we observed good agreement with our results at the energy of 6 eV. In contrast to the comparison with the benzene molecule, for angles above 105°, the ICS for the three systems (pyridine, pyrimidine, and pyrazine) exhibit a similar oscillatory behavior but with a small increase in the cross section. This effect may be related to the presence of the nitrogen atom in the ring structure, an element absent in the benzene molecule. For energies above 10 eV, there is excellent agreement among the curves for all of the molecular systems displayed in this figure. Finally, a possible explanation for the differences observed below 10 eV relies on the fact that, at this energy range, molecular characteristics play a significant role in electron-molecule scattering dynamics. In this region, the interaction time between the incident electron and the molecular target is long enough that the projectile perceives structural differences among the molecules. Conversely, for energies above 10 eV, the electron does not distinguish these variations in the molecular composition, leading to cross sections for the four systems considered in the comparison. The fact that, at these energies, the electron’s de Broglie wavelength is comparable to or smaller than the size of the molecules, preventing it from distinguishing the structural details of the molecular targets, is a possible rationale for this behavior.

**Figure 4:**
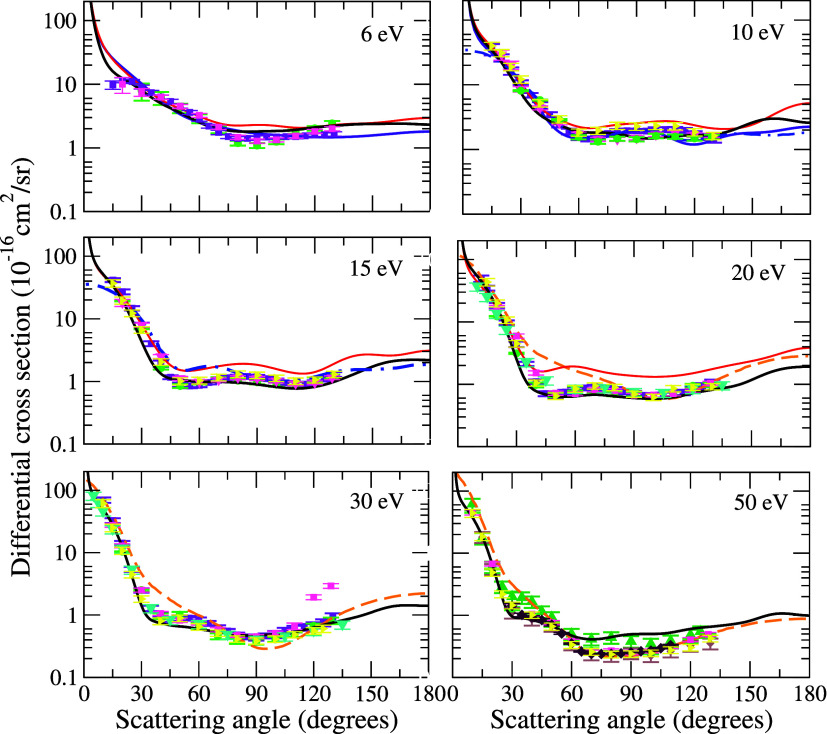
Differential cross sections for elastic electron scattering by pyridine at the impact energies of 6, 10, 15, 20, 30, and 50 eV. Black solid line, present results. Theoretical results: red solid line, cross sections obtained by Barbosa et al.^15^ using the SMC method with the Born-closure correction; orange dashed line, cross sections obtained by Costa et al.^16^ using the IAM+SCAR method; blue dashed-dotted line, cross sections obtained by Costa et al.^16^ using the R-matrix method; indigo solid line, cross sections obtained by Su et al. using the R-matrix method in the SEP+Born approximation. Experimental results for the benzene molecule: purple squares, measurements reported by Cho et al.;^48^ yellow triangle left, measurements reported by Cadena et al.;^47^ brown diamonds, measurements reported by Kato et al.;^50^ spring green triangles up, measurements reported by Sanches et al.^49^ Experimental results for the pyrimidine molecule: pink squares, measurements report by Palihawadana et al.;^53^ turquoise triangles down, measurements reported by Baek et al.;^51^ diamonds maroon, measurements reported by Maljković et al.^52^ Experimental results for the pyrazine molecule: green circles, measurements reported by Palihawana et al.^54^

### 4.2 Inelastic Cross Section

In Figure 5, we present the electronically inelastic integral cross sections corresponding to transitions from the ground state to the 1^3^ *A*_1_ (3.67 eV), 1^3^ *B*_2_ (4.57 eV), 2^3^ *A*_1_ (4.98 eV), 1^3^ *B*_1_ (5.61 eV), and 1^3^ *A*_2_ (6.82 eV) triplet states, as well as to the 1^1^ *B*_2_ (6.15 eV), 1^1^ *B*_1_ (6.45 eV), and 1^1^ *A*_2_ (6.94 eV) singlet states, induced by electron impact on the pyridine molecule for energies up to 10 eV. Our results are compared with the theoretical data obtained by Su et al.^17^ In general, the cross sections exhibit a similar behavior, differing mainly in magnitude, with our results being slightly higher than those obtained by Su et al.^17^ These authors characterized some of the structures observed in the cross sections using the phase-sum method. For the first two (1^3^ *A*_1_ and 1^3^ *B*_2_) triplet states, Su et al.^17^ identified a structure in the cross sections within the energy range of 5–6 eV, attributed to the 2^2^ *B*_1_ core-excited resonance observed in the elastic channel. We also observed a structure in the present ICS curve for the first triplet state (1^3^ *A*_1_), which may be associated with the resonance appearing in our ICS curve for the elastic channel at the energy of 5 eV. Furthermore, Su et al.^17^ also discussed the character of the structures found in the cross sections for transitions to the 1^3^ *B*_2_, 1^1^ *B*_1_, and 1^1^ *A*_2_ electronic states around 7 eV, attributing these to the influence of the core-excited ^2^ *A*_2_ resonance. Structures in the energy range of 7–8 eV, originating from the electronic states 1^3^ *B*_2_, 2^3^ *A*_1_, 1^1^ *B*_2_, 1^3^ *A*_2_, and 1^1^ *A*_2_, are associated with the core-excited resonance in the 4^2^ *B*_1_ symmetry. Finally, structures above 8 eV are attributed to threshold effects in the ionization channel.

**Figure 5:**
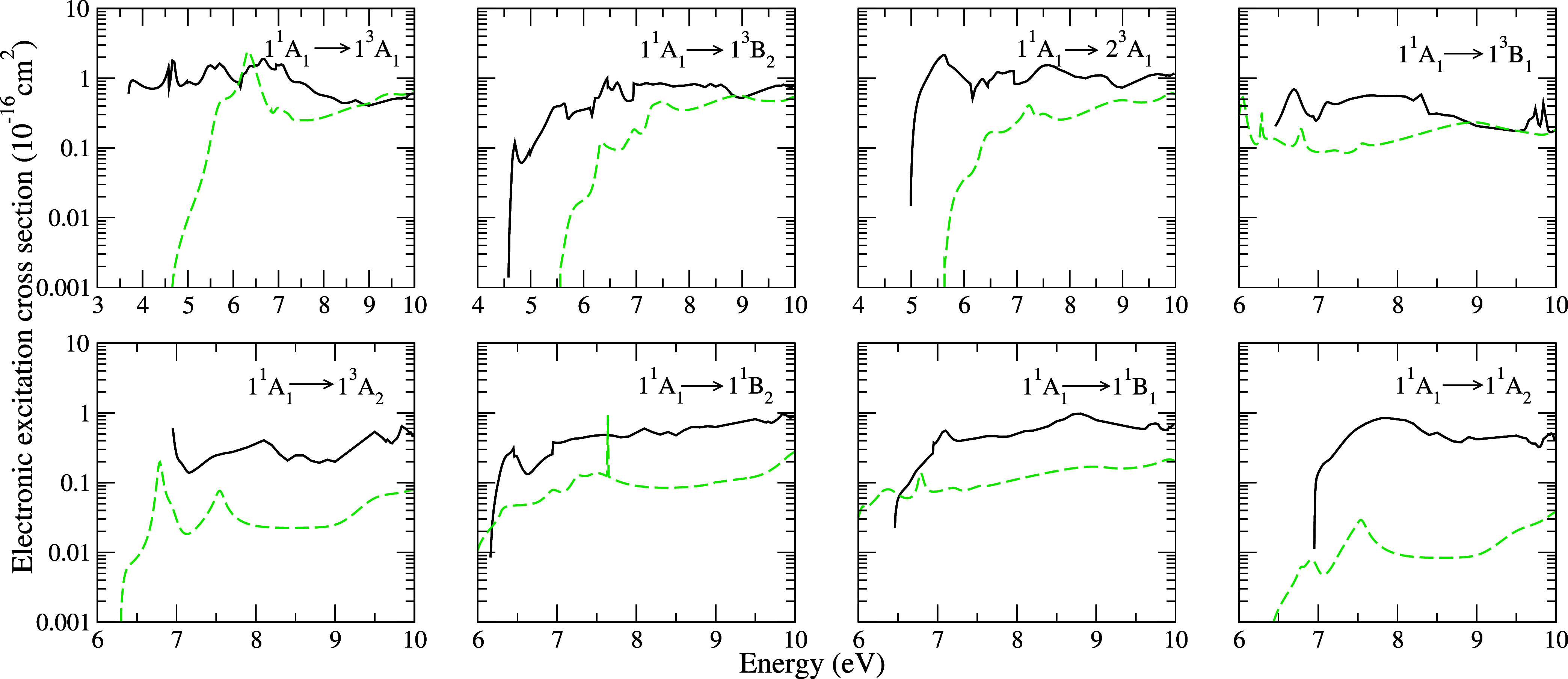
Integral cross sections for the excitation from the ground state to the 1^3^ *A*_1_ (3.67 eV), 1^3^ *B*_2_ (4.57 eV), 2^3^ *A*_1_ (4.98 eV), 1^3^ *B*_1_ (5.61 eV), 1^3^ *A*_2_ (6.82 eV), 1^1^ *B*_2_ (6.15 eV), 1^1^ *B*_1_ (6.45 eV), and 1^1^ *A*_2_ (6.94 eV) excited states of pyridine by electron impact. Solid black line, present results; dashed dark green line, theoretical results obtained by Su et al.^17^ using the R-matrix method in the CC approximation.

In Figure 6, we present the cross section for electronic excitation by electron impact with energies up to 50 eV, given by the sum of the contributions of the 300 electronically excited channels considered in the present calculation. Our results are compared with those of Costa et al.,^16^ obtained using the IAM+SCAR method. At low energies, our calculations yield magnitudes smaller than those reported by these authors,^16^ a discrepancy which may be related to differences in how each method describes the effect of flux stealing due to the contribution of inelastic channels in the scattering calculations. While the IAM+SCAR method employs an absorption potential to simulate this effect, the SMC method explicitly treats each excited state through the projection operator onto open channels. In the 10–20 eV range, our results show good agreement with those of the authors, whereas above 20 eV, they exceed them in magnitude.

**Figure 6:**
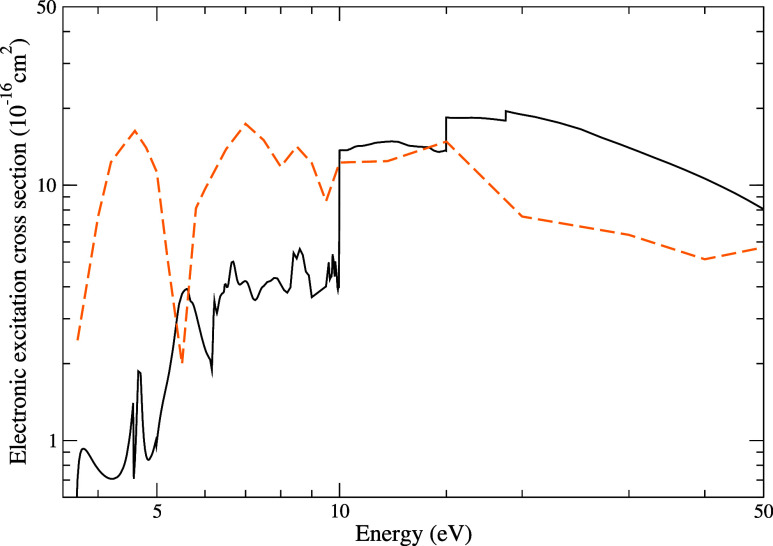
Cross section for electronic excitation by electron impact on the pyridine molecule, considering impact energies up to 50 eV. Solid black line, present results; dashed orange line, results obtained by Costa et al.^16^ using the IAM+SCAR method.

### 4.3 Ionization Cross Section

In Figure 7, we present our TICS for electron scattering by the pyridine molecule obtained using the BEB model for impact energies ranging from the first ionization threshold up to 1000 eV. We compare our cross sections with the theoretical results from Gupta et al.,^55^ who employed the spherical complex optical potential formalism and the ionization contribution method based on the complex scattering potential, and from Costa et al.,^16^ who used the IAM+SCAR method to determine the TICS. Additionally, we compare our results with the experimental data from Jiao et al.,^56^ obtained using Fourier transform mass spectrometry to investigate the dissociative ionization of pyridine by electron impact. Our results show good agreement with those obtained by Costa et al.^16^ and exhibit a similar trend compared to the values reported by Gupta et al.^55^ and the experimental data from Jiao et al.^56^ Although differences in magnitude are observed in comparison to the former two results, our TICS remains within the error margin associated with the measurements performed by Jiao et al. As expected, our TICS presents a maximum around 70 eV, decreasing as the energy increases, in excellent agreement with the behavior predicted by other results available in the literature. Despite the discrepancy in terms of magnitude, we conclude that our TICS provides a very reasonable estimate to account for the contribution of ionization to the sum of the processes comprised in the total cross section.

**Figure 7:**
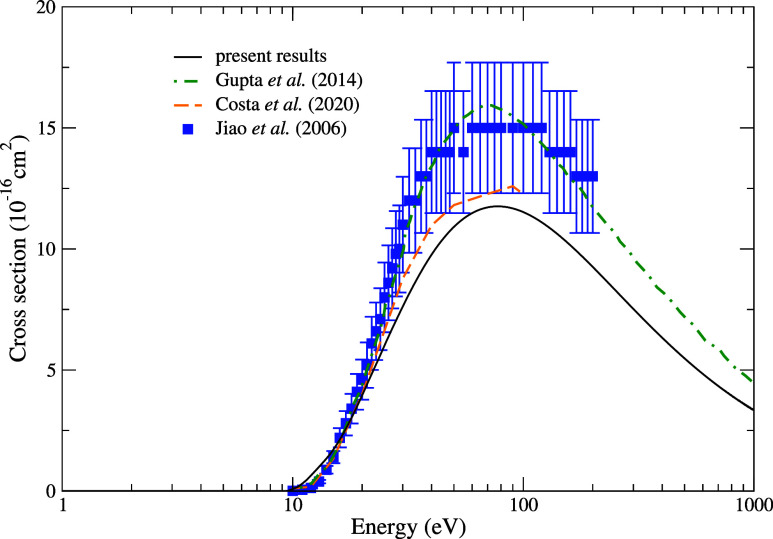
Ionization cross sections for electron scattering by the pyridine molecule. Solid back line, present results; green dashed-dotted line, theoretical results obtained by Gupta et al.;^55^ dashed orange line, theoretical results obtained by Costa et al.;^16^ blue square, measurements reported by Jiao et al.^56^

### 4.4 Total Cross Section

In Figure 8, we present our computed TCS. To estimate it, we summed the contributions from the elastic and electronically inelastic channels, obtained using the SMC method, along with the total ionization cross section contribution estimated by the BEB model. This approach to evaluate the TCS has been adopted by our group with satisfactory results so far.^6,25,26^

**Figure 8:**
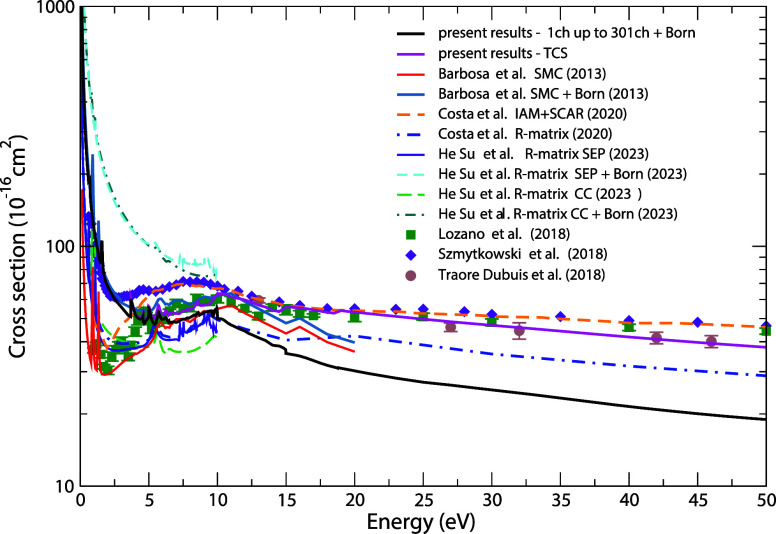
Total cross section for electron scattering by the pyridine molecule for impact energies up to 50 eV. Magenta solid line, present TCS, including elastic and electronically inelastic contributions obtained using the SMC method, plus the total ionization cross section contribution computed with the BEB model; TCS obtained by Costa et al.^16^ using the IAM+SCAR method; blue dashed-dotted line, indigo solid, electric blue long-dashed, spring green short-dashed, and electric blue double-dotted-dashed lines, TCS obtained by Su et al.^17^ using the R-matrix method in the SEP, SEP+Born, CC, and CC+Born approximations, respectively; green squares, experimental results from Lozano et al.;^19^ purple diamonds, experimental results from Szmytkowski et al.;^20^ and brown circles, experimental results from Dubuis et al.^18^

As can be seen, for energies between 5 and 20 eV, our cross section is in good agreement with the result obtained using the IAM+SCAR method by Costa et al.^16^ For values just above 20 eV, the result from Costa et al.^16^ becomes slightly higher than ours but still exhibits the same overall behavior. For energies below 10 eV, however, there is a discrepancy between the results, which is expected since the IAM+SCAR method treats atoms as independent, whereas in this energy range, molecular effects become significant. Su et al.^17^ presented the TCS obtained using the R-matrix method at two levels of approximation, SEP and CC, both with and without the Born-closure correction. Compared to our TCS, the R-matrix results that include the Born correction overestimate our cross sections at low energies. However, as the energy increases, the magnitudes of the cross sections begin to converge, showing reasonable agreement for energies around 10 eV. Regarding the comparison with the experimental TCS data, our results show excellent agreement with the values reported by Lozano et al.^19^ in the 5 to 30 eV range. However, for energies below this interval, the agreement is not as satisfactory. This discrepancy can be attributed to the angular resolution of the apparatus used in these measurements, which has a strong dependence on the energy. At lower energies, the results are more sensitive to the angular resolution, leading to an underestimation of the actual TCS values in the measurements. With regard to the TCS obtained by Szmytkowski et al.,^20^ we observe excellent agreement at low energies, where the abrupt increase is attributed to the interaction with the long-range dipole potential. In the 3 to 10 eV energy range, there is a significant discrepancy in the magnitude of the cross section, with our results lying below the values reported by these authors, possibly due to the presence of threshold effects and core-excited resonances observed in our cross section in this energy range. Above 10 eV, the TCSs show reasonable agreement, exhibiting similar behavior. The TCS data obtained by Traoré Dubuis et al.^18^ exhibit excellent agreement with our results across the entire energy range here considered. Overall, our results show good agreement with the experimental data available despite some differences mentioned above.

## 5 Conclusions

We presented elastic and electronically inelastic cross sections for electron scattering by the pyridine molecule obtained by using the SMC method. Additionally, we calculated the ionization cross section using the BEB model and estimated the total cross section by summing the elastic and inelastic contributions obtained with the SMC method with the ionization cross section provided by the BEB model. Our calculations take into account the effect of multichannel coupling, considering from 1 channel (elastic channel) up to 301 open channels, a channel coupling scheme defined according to the MOB-SCI strategy.

We compared our results for elastic scattering with the theoretical and experimental data available in the literature. The positions of the resonances are well described, and the assignments for their character are consistent if compared to those reported in previous studies. Our elastic ICS is in reasonable agreement with other theoretical results below 10 eV. Above this energy, the magnitude of the present cross section displays a significant reduction as a result of the flux stealing, owing to the competition among the elastic and all electronically inelastic channels included in our calculations. Inclusion of multichannel coupling effects in the description of elastic electron scattering by pyridine provides elastic DCSs that are in excellent agreement with the experimental data available for systems with molecular structures similar to those of pyridine. Although such an agreement provides an indication of the importance of these effects, experimental measurements involving the pyridine molecule would still be welcome to confirm this finding.

Regarding electronic excitation, we compared the transition cross sections from the ground state to five triplet and three singlet states with the available literature data. We obtained some relevant insights, observing similarities in certain structures of the cross sections. We also compared the total electronic excitation cross section, considering all excited states included in our calculations, with the literature data, finding some agreements. However, the problem of electronic excitation remains open, highlighting the need for further studies and, in particular, more experimental data to better understand this process. We also presented the integral cross sections decomposed by symmetry and discussed the origin of the structures observed in the integral and total cross sections below 10 eV, relating them to the transition cross sections for the first six triplet states and the first four singlet states.

The total cross section estimated in our work shows excellent agreement with the theoretical and experimental results reported in the literature. In contrast, the electronic excitation cross sections exhibit discrepancies with the available data, highlighting the need for further investigations to elucidate the origins of these divergences.

Pyridine serves as a toy model of one of the simplest and most representative molecules for studying the effects of radiation on the nitrogenous bases found in DNA and RNA. Due to its relevance, it is an excellent candidate for both theoretical and experimental investigations involving interactions between charged particles and biomolecular systems. In this study, we provide a detailed set of cross sections—including elastic scattering, electronic excitation, and ionization processes, which can offer essential input for simulations and modeling in biologically relevant molecular environments. The results presented here have the potential to significantly enhance the databases used in research on the mechanisms of radiation-induced molecular damage.
